# Pharmacological limitations of phage therapy

**DOI:** 10.1080/03009734.2019.1688433

**Published:** 2019-11-14

**Authors:** Anders S. Nilsson

**Affiliations:** Department of Molecular Biosciences, The Wenner-Gren Institute, Stockholm University, Stockholm, Sweden

**Keywords:** Bacteriophage, phage therapy, pharmacodynamics, pharmacokinetics, pharmacology

## Abstract

Clinical trial results of phage treatment of bacterial infections show a low to moderate efficacy, and the variation in infection clearance between subjects within studies is often large. Phage therapy is complicated and introduces many additional components of variance as compared to antibiotic treatment. A large part of the variation is due to *in vivo* pharmacokinetics and pharmacodynamics being virtually unknown, but also to a lack of standardisation. This is a consequence of the great variation of phages, bacteria, and infections, which results in different experiments or trials being impossible to compare, and difficulties in estimating important parameter values in a quantitative and reproducible way. The limitations of phage therapy will have to be recognised and future research focussed on optimising infection clearance rates by e.g. selecting phages, bacteria, and target bacterial infections where the prospects of high efficacy can be anticipated, and by combining information from new mathematical modelling of *in vivo* pharmacokinetic and pharmacodynamic processes and quantitatively assessed experiments.

## Introduction

The ability of bacteria to develop resistance against antibiotics is probably as old as the bacteria themselves and has been a concern ever since the introduction of the antibiotics we use today (1). However, the current overuse of antibiotics has led to an accelerating spread of antibiotic resistance, and there is no development of new antibiotics taking place (2). The use of bacteriophages, i.e. phage therapy, for the treatment of bacterial infections is not a new idea but has gained attention over the past 20 years as a possible alternative treatment method due to the emergence of antibiotic-resistant bacterial strains (3). The gained interest has mainly been fuelled by the fact that phages can be shown to specifically kill almost any bacteria, are easy and cheap to isolate, and do not interfere with normal human bacterial flora nor the environment (4,5). However, in spite of the need for new ways to cure bacterial infections, and a long history of trials, clinically applied phage therapy is not routinely carried out. There are probably two main reasons for this. Firstly, phages are very different from conventional antibiotics. They can obviously kill bacteria, but that does not necessarily imply that they can be used therapeutically; phages are host strain-specific and have special pharmacokinetics (PK) and pharmacodynamics (PD) which demands that methods, from isolation to clinical use, will have to be developed and tailored to each individual phage–bacteria combination (see (6,7) for a definition of phage therapy PK and PD). The PK and PD of phages applied *in vivo* are, however, poorly understood and not part of the current research agenda. Secondly, the long history of using conventional antibiotics has led to the establishment of socio-economic structures and drug regulation policies which taken together makes it virtually impossible to establish phage therapy. In other words, the push from the scientific community is too weak, with no comprehensive studies demonstrating a sufficiently strong and clinically relevant result of phage therapy that could motivate continued development. Likewise, the pull from society and pharma industry is equally weak to make huge investments in a completely new way of treating bacterial infections without relevant proof of concept, and with major regulatory problems.

The deadlock is presumably not going to be broken by more *in vitro* studies of particular phages being effective in killing a certain strain of a pathogen, or by the results from well-designed murine infection models (even though these have contributed substantially to the understanding of the complexity of phage therapy pharmacology). Moreover, there are far more economically interesting pharmacological research and development projects for the pharma industry to get involved in. What is needed is a number of clinical trials showing a generally high level of infection clearance comparable to antibiotics and significantly higher than in the trials conducted so far.

## Clinical trials of phage therapy

**Figure 1.**
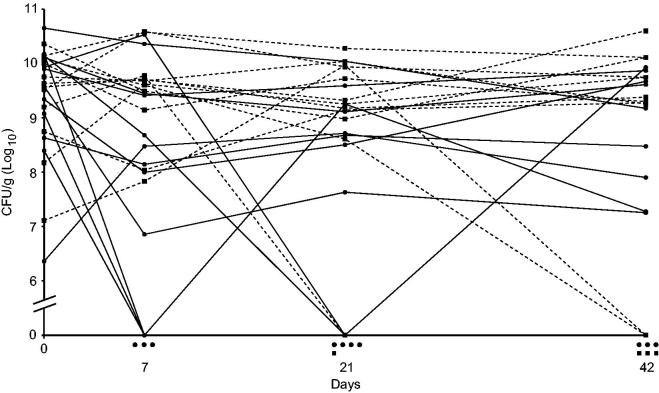
A randomised double-blind clinical trial of phage therapy against *Pseudomonas aeruginosa* otitis media. Patients were divided into two groups, one treated with phages (solid lines, ●) and the other with placebo (dashed lines, ■). There is a positive effect of the treatment in three patients as shown by non-detectable counts of the bacterium (CFU/g) after 7 days, but the infection remains in the majority of patients as in most of the patients treated with placebo. Symbols under the *x*-axis indicate the number of patients in the two groups with non-detectable counts of *P. aeruginosa* at the different time points. Data from (14).

The outcome of clinical trials with different phage–bacteria combinations is heterogeneous, as the variation among trial participants ranges from complete clearance of bacteria to no effect at all (8–10). Reports of randomised and double-blind clinical trials that have been carried out discuss that the reason for phage treatment failure could be the complexity of intestinal bacterial infections due to other co-infecting bacteria (11), or, in the case of burn wounds, technical difficulties including interfering treatments with antibiotics or too low titres of phages or target bacteria (12,13). Another careful trial reports a reduction of the mean of *Pseudomonas aeruginosa* counts in the outer ear of chronic otitis patients after 1, 3, and 6 weeks, as compared to placebo treatment (14). The infection was cleared in 3 out of 12 cases but remained in the other cases with only a minor reduction, or even increase, of bacterial counts (Figure 1). Apparently, phage therapy worked in some but not all cases, and the average reduction mainly depended on the cases where the therapy worked. Possible explanations to the varying results in this case may be the very low dose of phages applied, 2 × 10^4^, of each phage in a cocktail, development of phage resistance, or variation in obstructive biofilm formation, which is a known problem with *P. aeruginosa* infections (14).

The largest and possibly most comprehensive report of clinical phage therapy in general, from the Phage Therapy Unit in Wrocław, Poland, shows inconclusive results. The patient infection status after treatment was assessed and classified into seven categories. Patients in categories A, ‘pathogen eradication’, B, ‘good clinical result’, and C, ‘clinical improvement’, were considered to be good results of the treatment and constituted 40%, whereas categories D, ‘questionable clinical improvement’, E, ‘transient clinical improvement’, F, ‘no response to treatment’, and G, ‘clinical deterioration’, constituted 60% of the patients (10). However, the trials were not randomised, double-blind, clinical trials, and the report involves many different types of infections, bacteria, phage preparations, and treatment procedures. The overall infection clearance rate was, however, significantly lower than previously reported by the Polish researchers (15,16). This difference is explained by adopting more stringent assessment criteria of treatment results (10).

Taken together, the results of clinical trials of phage therapy clearly show a broad variation in efficacy, which makes it very difficult to predict the outcome of a treatment in individual cases. However, many smaller trials and single compassionate treatments have been carried out lately (17,18). Phage therapy of single patients is occasionally effective, but due to the severity of their infections, simultaneous antibiotic therapy is common, and negative controls are naturally missing. There are also many preclinical and a few clinical phase 1–2 trials being planned to start soon (19).

## Phage therapy research

Phage therapy as a field of research has been dominated by experimentalists, and the majority of the experiments referred to above were carried out without much phage therapy-specific theoretical consideration, applying a phage or phage cocktail that had been proven to be highly effective *in vitro*. Such *in vitro* efficacy can be delusive (e.g. if based on qualitative spot test screening only (20)), and planning of the experiments and assessment of results were subsequently focussed on the overall efficacy of the treatments and not on how, or why, it worked or not. As a consequence, pharmacological data from experiments are more often than not missing, and values of the many parameters that affect the outcome of phage therapy still remain unknown.

Phage therapy theory, on the other hand, is mostly based on results from mathematical models of phages infecting bacteria in bioreactors or chemostats (21–24). These are mostly mass-action-based models that consequently reduce the probability of infection to be dependent on the titre of free phages, the uninfected bacteria, and the adsorption rate only; spatial distribution of phages and bacteria is considered to be uniform, and the diffusion rate infinite. Phage biologists have, however, studied phages–bacteria infection dynamics for a long time, and the values of many *in vitro* infection parameters—e.g. adsorption rate, latency times, and burst sizes—of many phage–bacteria combinations are known. Efforts have been made to modify mathematical models, and encompass more and other infection parameters to more closely fit actual conditions, including e.g. better simulation of adsorption dynamics, biofilm formation, and distribution of latency time and burst size (24–26, and references therein). As such, the models do not completely reflect the pharmacology of phage therapy, but they can nevertheless contribute with data on some parameters, and their relative importance, albeit under idealised conditions. Current PD models can become the foundation for new refined models with the addition of *in vivo* data, but models of PK based on *in vivo* data must also be developed and incorporated into new mathematical models.

## Phage therapy pharmacological complications

The aim of phage therapy is to maximise the number of phages that reach and infect as many bacteria as possible, and that these phage infections eventually result in clinically insignificant levels of bacteria without causing unwanted side effects. In order to accomplish this, not only the titre of phages but also the titre of the bacteria must be sufficiently high at the site of infection, and the phage titre must pass the ‘inundation threshold’ where the phage replication outruns the bacterial replication (27,28). This can be achieved either through a single phage infection cycle or by the following cycles of phage infection and reproduction (i.e. active or productive phage infection), but also by repeated administration of phages. It may seem counter-intuitive, but the effectiveness of phage therapy increases as the concentration of bacteria increases. The likelihood that a phage hits a bacterium increases as does the production of more phages. Looking back at the results from phage therapy experiments and clinical trials, it seems that the unpredictable or poor results in many cases are due to the combined effect of the PK properties of phages and the *in situ* PD of phages–bacteria. Phages have been applied clinically without recognising the full complex dynamics arising from interactions between the human body, bacteria, and phages, and the quantitative data that would allow reproducing the trials are often missing (29,30).

### Phage pharmacokinetics

The unusual PK is a consequence of phage particles being a million times larger than any antibiotics molecule, and consisting of several different proteins. Their size limits the dose that can be given as well as lowers the uptake and transportation rates, and their protein nature causes them to be eliminated by the mononuclear phagocytic system (31). In comparison, low-molecular-weight antibiotics have far better properties. Ofloxacin, a common broad-spectrum fluoroquinolone antibiotic, reaches a concentration of around 2 µg/mL in serum after a standard oral dose of 200 mg, equivalent to about 3 × 10^15^ molecules/mL, with a half-life in plasma of around 30 min, and 12 h in tissue (32,33). In comparison, phages are distributed and taken up by most organs regardless of route of administration (34, and references therein), but the uptake is lower and hence also the titres in different tissues. As a consequence of the size of phages, a phage suspension cannot contain more than approximately 10^13^–10^14^ plaque-forming units/mL (PFU/mL). It is, however, technically difficult to achieve more than 10^11^ PFU/mL crude lysate, and the titre is further reduced after purification (5).

There are no comparable human studies on the phage titre in plasma following oral administration of phages, but a human phage therapy safety test reports a peak average of 3 × 10^4^ PFU/mL in stool samples after drinking mineral water supplied with 10^5^ or 10^7^ T4 phages for 2 days, according to different schedules (35). It can, however, be assumed that phages are taken up and transported similarly in other vertebrates (36), and PK of phages have been studied in many animal models (37,38). The results from a rat model showed an increase in PFU in six organs after intraperitoneal or subcutaneous injection of 10^8^ PFU in rat pups. The phage titre was about 10^7^ PFU/mL in blood 2 h after intraperitoneal injection and dropped to below 10^4^ PFU/mL after 24 h. The concentration of phages in spleen and kidney was, after the same time, above 10^6^ PFU/g (37). A study of phage PK in rat reports an overall PFU/mL of a phage cocktail over 10^8^ in serum after a 1 mL intravenously administered bolus of 10^10^ PFU/mL, and a decline down to 10^5^ PFU/mL after 24 h, indicating an elimination half-life of about 2.3 h (39). Continuous infusion of 0.1 mL/h for 24 h of the same phage cocktail and titre resulted, however, in a serum concentration of 10^7^ PFU/mL. The rate of clearance of phages caused by neutralisation by antibodies, and subsequent phagocytosis, lowers the titre (40–42) and affects the efficacy of a treatment when phages are administered during a longer period of time, but the loss differs between different phages.

Another reason for inefficient outcome of phage therapy might be a tendency for phages to bind to bacterial debris resulting from already lysed bacteria, blocking their tail fibre receptor-binding protein, and preventing them from adsorbing to live bacteria (43,44). This is not studied empirically in detail in the context of phage therapy, but there is an indication that phage titres from the second and following cycles of phage infection become unexpectedly low in *in vitro* experiments (45,46). However, there are many different structures that phages potentially can bind to, and the interference with other structures, e.g. exopolysaccharides, may play a more significant role for inactivation of phages. The influence on the infection kinetics from inactivation factors, as well as the importance of uneven distribution of cells and phages, e.g. as would be the case with biofilm formation, has been investigated in models by Bull et al. (47). Phages may also exhibit differences in their propensity to bind to cells of different tissues, which in some cases may lead to an even greater loss of phage titre, but it has been hypothesised that this can be turned into an advantage as it might be utilised for homing of phages to particular tissues (48).

The phage dose reaching the site of infection can thus be assumed to be substantially lower than the given dose in the majority of phage treatments, and rapidly drop to even lower levels if phages are not added continuously. This does not necessarily mean that a treatment will fail, since the success of phage therapy is a matter of probability that relies on many factors, e.g. the density of both phages and bacteria and the phage adsorption rate, but the probability that it does fail increases. A low dose of phages will require that a sufficient number of bacteria get infected (which can happen if the bacterial density is high enough), that the phages produced can spread to all infected sites, and that the rate of phage amplification is higher than the bacterial growth rate at particular sites of infection (28,49). The last-mentioned leads to the importance of also understanding phage infection PD *in vivo*.

### Phage pharmacodynamics

Recognising the shortcomings of phage therapy due to their special PK properties mentioned above, it can be assumed that the explanation for phage therapy being occasionally successful is phages’ ability to replicate and compensate for a low dose. Different mathematical models and experiments with phages–bacteria, in batch or continuous cultures, have contributed to the understanding of their kinetics during *in vitro* infection (21–23,45,50–53). With basic mathematical models, it is easy to show that any virulent phage with ordinary infection characteristics will completely eliminate all bacteria if the titres of phages and bacteria are sufficiently high and if the bacteria are not allowed to become resistant (50). On the other hand, infection experiments e.g. in chemostats in most cases show something different; after an initial dramatic decline, bacteria grow back to high titres, and the phages are often maintained at high titres as well. The bacteria appear not to become uniformly resistant, and there must be enough susceptible bacteria to be able to sustain a large phage population. Mathematical models and other experiments, including experiments with periodic nutrient supply, seem under many conditions also to result in stable coexistence of bacteria and phages (21,54). There could be many reasons for this, one being the obvious development of bacterial phage resistance through acquired mutations of receptor genes or by regular genetic systems (55,56), followed by counter-mutations by the phages and a continued and endless arms race. However, a high rate (10^−5^ per cell per hour) of genetic transition from resistant to susceptible cells has also been observed, without this being explained by host range mutations in the phage population (57).

A bacterial population is not a homogeneous collection of uniform and equally susceptible cells. Apart from cells being genetically different, i.e. carrying different mutations of which some may confer complete or partial resistance, the susceptibility to infection and ability to produce phages may also show spatial and phenotypic variation upon infection.

Spatial heterogeneity in a growing culture may arise from bacteria protecting themselves in surface biofilm or hiding in crevices in the vessel wall from where they can regrow once the phage titre is low enough to permit it (24,58). *In vivo* dynamics, i.e. titres of phage and bacteria during phage therapy, is probably even more complicated as bacteria will have better chances of producing biofilm in a more diverse environment, or hide deep in tissue or intracellularly, and avoid getting infected. Susceptible bacteria will not be evenly distributed, their growth will be affected by nutrient availability, and phages will be more concentrated around lysed bacteria. As a consequence, there will be local differences in growth of bacteria and phages and accumulation of biofilm, released debris, and metabolites that may further impede phage replication, and the PD is also affected by the bacteria’s ability to form micro-colonies or other arrangements (7,47,59).

Phenotypic variation between individual cells of bacteria, and the physiological state of the bacteria, may also affect the probability of infection and the number of released phages from lysed bacteria (59,60). Phenotypic variation in susceptibility between cells may arise due to phase shifting between different states, most often resulting in changes in cell surface structures (61). While the frequency of such random shifts may be small, selection by phages would rapidly increase the number of non-susceptible cells and the population would eventually recover. If the shift varies at random between cells, and is genetically inheritable, an equilibrium between phages and bacteria would eventually occur. Other phenotypic changes affecting cell surface structures may be explained by epigenetic gene regulation events. DNA methylation is likely to be involved in the regulation of expression of certain pili and outer membrane proteins which potentially can act as phage receptors (62, and references therein). It is also possible that some bacterial receptors vary naturally in such a way that the rate of adsorption will vary in the population and allow selection of bacteria with a low adsorption rate, leading to the coexistence of bacteria and phages (59).

After successful adsorption by a phage, and when its nucleic acid has been introduced into the cell, bacteria may also show phenotypic variation in the capacity of replicating the phage. Individual bacteria can be dormant depending either on limited access to nutrition or random fluctuations between cells in the expression of key genes. Depending on the availability of nutrition, bacteria may lie dormant as spores or have their metabolism running low or completely turned off. It has been shown *in vitro* that, when infecting bacteria with phages under low-nutrition conditions, some cells will produce just a few phages and that phage production increases significantly after the addition of nutrients. A fraction of phage-infected cells can either be hibernating or be in a pseudolysogenic state when starved, but this is also dependent on the infection biology of the phage. Bryan et al. (60) showed that phage T4 can ‘scavenge’ on available resources and produce a small number of phages, but, in most infected cells, T4 hibernates and does not carry out the final steps of DNA degradation and subsequent reproduction until nutrients are added. Phage T7, in contrast, is less dependent on the nutritional status of the host bacteria. It has a smaller genome which requires fewer resources for reproduction and is adapted to take advantage of the resources readily available for its DNA replication and production of structural components (60,63). Pseudolysogeny of virulent phages, i.e. a stalled development which does not rule out a later full lytic cycle, may happen under nutrient-limited conditions but also when bacteria, having special growth requirements affecting their metabolism (e.g. pH, temperature, or salinity), grow under non-optimal conditions (64,65). Superinfection, the infection of an already phage-infected cell by the same or in some cases unrelated phages, can also fail as a result of the inability of the second phage to inject its DNA, i.e. superinfection exclusion, or it may lead to an extension of the length of the infection period, i.e. lysis inhibition (66–68).

Phage therapy using phage cocktails have been reported in a number of studies (13,69). There are two main reasons for simultaneous use of more than one phage against a bacterial infection. Firstly, when the bacterial strain and its susceptibility for phages are known, the phages in a cocktail can be chosen to infect the target bacteria by different receptors which likely will reduce the probability of development of resistance. Secondly, when the infecting bacterial strain and sensitivity to different phages is unknown, at least one phage in the cocktail may be able to infect. However, the composition of cocktails into liquid formulations limits the individual titre of each phage due to physical reasons, with the result that an already low dose of the phage or phages being potentially effective becomes even lower. Phage cocktails also bring additional complexity to the PD. Co-infection with two or more phages can in some combinations result in synergism, e.g. when one phage releases an enzyme that depolymerases biofilm, facilitating the infection by another phage (70), but phages competing for resources, as would be the case when more than one phage is able to infect the host, will in most cases interfere with one another. During co-infection, the phage with the highest adsorption rate, shortest latency time, and largest burst size will inevitably have a selective advantage, and slower phages will be eliminated; the result would be equal to an infection with a single phage at a lower titre. Furthermore, co-infection can also result in superinfection exclusion or lysis inhibition already mentioned, but also in cross-resistance where the resistance developed under infection of one phage results in resistance against other phages as well. Hence, the bacteria can become resistant to all phages infecting by the same receptor through a single mutation altering the structure of that receptor molecule, or against phages utilising different receptors by mutations affecting a common global regulator of multiple receptors (71).

Bacteria appear to have both genetically controlled resistance systems as well as regular phenotypic ‘contingency plans’ to quickly cope with phage infections. The extent of the various types of resistance in different bacteria and the reason for low efficiency, especially of the mechanisms described above, must be investigated more closely in order to understand the PD of phage therapy. Most mathematical models and *in vitro* experiments do not reflect all aspects of the interactions between phages and bacteria and are not particularly true to a real phage therapy treatment, as the majority of models are presuming phages and bacteria to be planktonic and the phage infection to follow mass-action kinetics. More recently, however, models have been developed which take into account some of the pharmacodynamical problems associated with the low efficacy of phage therapy. Recent theoretical models have for instance been developed that include the complexity arising from biofilm or spatial heterogeneity (47), as well as bacteria expressing ‘leaky resistance’ where a fraction of the resistant bacteria reverts to susceptibility at a high rate in spite of being surrounded by virulent phages (57), but every phage–bacteria combination is indeed different and general models may prove to be very challenging to construct.

## Future research and development

The *in vivo* PK and PD of phage therapy, and hence the outcome, cannot be anticipated from phage–bacteria *in vitro* infection experiments. There is a need for new models, animal experiments, and clinical trials reflecting genetic and phenotypic changes of bacteria and phages during *in vivo* phage therapy. The outcome of phage therapy trials at present varies from one individual to another and between different studies in such a way that the results are not reproducible. The aim of optimising is to eventually make the result of a phage treatment predictable for a large part of treated infections. In the first place, it will become necessary to focus the efforts and reduce the variation by limiting the number of phages, target bacterial strains, and infections to a few type cases. Secondly, the important parameters during *in vivo* experiments or clinical trials must be quantitatively assessed under different conditions to make it possible to get an idea of the importance and contribution of different components of variance and allow statistical analysis. Phage therapy experiments are generally evaluated at different endpoints and by qualitative, or semi-quantitative, measurements, but the optimisation of the efficacy of phage therapy is completely dependent on a systematic generation of quantitative *in vivo* data (29).

### Selection and basic characterisation of phages

In general, reducing the components of variance of phage therapy is theoretically easy but in practice more difficult to do because of the huge variation of phages and bacteria. While there are no virulent phages found for some pathogenic bacteria, e.g. *Clostridium difficile* and *Helicobacter pylori*, virulent phages can be isolated for most pathogens, but the number of phages needed to cover the variation among strains within a particular bacterium varies. It is essential that the phage not only possess good antibacterial properties against the bacterium, but equally important is that no gene products are expressed that add to unexplained variation and unwanted side effects. Firstly, there are genes with unknown function in virtually every phage genome. Genome sequencing data could reveal not only if a phage is truly virulent, which is desirable, but could possibly also rule out phage gene products that may be harmful to man or compromise a treatment (72–74). Secondly, phages may exhibit surface proteins that evoke the innate immune system as well as cause elevated levels of antibodies (40,41). Phages are, however, part of the microflora that we are exposed to in everyday life, the immune response varies from phage to phage depending on tissue, and it should be possible to find phages which do not give rise to, or only cause insignificant, immunological responses (42,75). However, it is difficult to compare the outcome of studies of immunogenicity originating from the phages themselves as there are many methods for purification, each leaving trace amounts of different fractions of bacterial debris from shattered bacteria which may confound the assessment. Reducing the variation coming from differences in immune response would require that phages that are going to be used in clinical trials be produced meeting the requirements set by international regulatory bodies for medicines. There is, however, currently a lack of a common best practice and standardised purification methods, and the degree of purity varies between methods (76,77). The best purifying method would need to be scaled up for fast production of larger volumes, and the phages would in most cases need to be formulated into stable medical preparations without reducing phage titres or phage function. Phages can also be selected *in vivo* for improved stability and extended circulation time by e.g. mutation selection, or formulated by encapsulation, lyophilisation, or PEGylation (78–82). Many other formulation strategies, e.g. nebulisation or freeze-drying, have also been elaborated that have resulted in greater stability and higher titres at the infection site (9,83).

Phages’ suitability for phage therapy should be quantitatively shown in efficiency of plating (EOP) analyses and not merely in spot tests, since the latter may result in false positives and overestimation of the host range (20,84). The high efficacy of the phage on the bacterial strain should also be reproducible *in vitro*, with insignificant variance between experiments. That would in turn indicate absence of defence mechanisms against the actual phage, bacterial phase or antigen shifting, masking or sporulation, as well as a low rate of development of any type of resistance against the phage. Induction of prophages, or genes within prophage genomes, may also contribute to unwanted variation. This may seem like a benefit, as the bacterial host cell lyses and the released temperate phages can continue to infect other bacteria, but overall this can have the opposite effect; as the number of hosts decreases, the efficacy of the virulent phage goes down and bacteria containing the prophage would be immune to superinfections by the same phage. Interference with an induced prophage would not matter if the rate of induction is completely predictable, but it may be triggered by unknown and varying environmental factors and cause random variation. It is well known that prophage genes that get induced interfere with infecting virulent phages; expression of non-essential genes in prophages have been shown to block other phages from replicating, and are often constitutively transcribed from prophages in the bacterial genome (85). Prophages are also frequently encoding bacterial virulence factors that can be induced by unknown factors, possibly including infection by virulent phages (86,87). In addition, the bacterial strain should preferably not produce exotoxins and only produce small amounts of endotoxins released upon lysis, and bacteria causing intracellular infections must of course also be ruled out.

### Optimising the pharmacokinetics and pharmacodynamics

The pharmacokinetic parameters that primarily influence the outcome of *in vivo* phage therapy are the phage titre (dose and dosage administered), delivery routes, pharmaceutical formulation, phage adsorption rate, phage decay or elimination rate, and the rate of diffusion or transportation of phages. Most of these parameters have not been subjected to optimisation, and it must be pointed out that the efficacy of phage therapy is highly dependent on the pharmacodynamics too. Phage therapy may fail even if the pharmacokinetics is optimised, e.g. a very high single dose of phages will be in vain if the bacterial concentration, movement of phages and bacteria, or the adsorption rate is too low. Although this may lead to a reduction of bacterial titres, it will not lead to productive phage infection (only to a local increase in phage titre) and subsequent spread of phages, and the bacterial population would consequently continue to grow after the phages have been degraded or eliminated.

**Figure 2.**
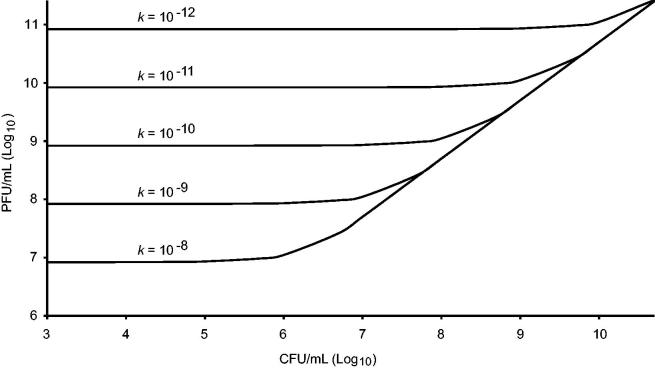
Phage titre needed to reach an actual multiplicity of infection (MOI_actual_) of five after 1 h of infection at different adsorption rate constants (*k* = adsorbed phages/mL min), and as a function of the bacterial titre, in an ideal pelagic system. At MOI_actual_ = 5, the probability that a bacterium gets infected is 0.99. The functions converge to the diagonal line when MOI_actual_ = MOI_input_. For example, at a CFU/mL of 10^8^, a MOI_input_ of 5 equals MOI_actual_ if the adsorption rate is higher than 10^−9^, but if it is lower than 10^−12^ a MOI_input_ of about 1000 (10^11^ PFU/mL) is needed. Function values are constant at lower CFU/mL where MOI_input_ has to be substantially higher especially if the adsorption rate is low.

A phage dose as high as it can possibly be, without inducing the immune system, would be a good start when optimising a phage treatment, but the phage adsorption rate has a large influence on the efficacy too and has to be considered. Under ideal conditions, assuming constant mixing of planktonic cells and phages, the number of phages that actually adsorb and infect a bacterium follows the Poisson distribution. As the mean probability of infection goes down, the number of uninfected bacteria increases. This has led to the concept of *actual* multiplicity of infection (MOI_actual_) in contrast to the added MOI_input_ (MOI_input_ = concentration of phages/concentration of bacteria):

$${\text{MOI}}_{\text{actual}}=\left(1-e^{-\mathrm{kCt}}\right){\text{MOI}}_{\text{input}}$$

where *k* is the adsorption rate constant, *C* is the concentration of bacteria/ml (CFU/ml) of bacteria and MOI_actual_ the number of phages bound to bacteria at time *k* (Figure 2). At low titres of bacteria and a low adsorption rate, it may take a long time for the maximum number of bacteria to become infected even though a high titre of phage is added (49,88).

Maintaining a high titre is especially important if the adsorption rate is low. Phages might otherwise become degraded before they can adsorb to the bacteria. To optimise phage therapy, knowing the adsorption rate under the conditions that apply during treatment is essential, and the best alternative would be to choose phages with a very high adsorption rate (for instance T1-like phages: T1 itself has an adsorption rate of 3 × 10^−9^ ml × min^−1^ (89)). *In vivo* phage adsorption rates are, however, affected by the movements of both phages and bacteria, and a high *in vitro* adsorption rate alone does not guarantee phage therapy success. However, small phages might be better than large phages in terms of diffusion and mobility *in vivo*. In addition to the fact that small phages should have a higher diffusion and transportation rate, some large phages can be disadvantageous as they may have protrusions that can bind to mucus layers, which reduces their mobility (30). It has been suggested that binding to certain cell receptors could be turned into an advantage by adding surface epitopes to the phage, causing improved affinity for a certain tissue and a homing of phages to infected tissues (48).

Another way to solve the phage-dosing problem is to supply phages in repeated high doses and sustain a high titre over a long period of time (39). This, of course, requires that the phages do not give rise to any serious immunological reaction (which is the main explanation to why the efficiency of phage therapy need to be optimised, not maximized). From a pharmacodynamic perspective, the latency time should be short and the burst size large, but it is of equal importance that the phage infection is independent of the nutritional status of the bacteria and that the phage does not enter pseudolysogeny or causes superinfection exclusion, lysis inhibition, or other obstacles for replicating. All of these parameters can be assessed *in vitro* as well as the propensity for development of phage resistance or induction of inhibiting genetic systems, taking into account that these properties may very well be observed only under ideal conditions and not reflect what is going to happen *in vivo*.

Are cocktails better than a repeated dose of the same or alternatively different phages? Cocktails may have advantages when the infecting bacteria are unknown or to delay the development of resistance of the infecting bacterial strain, but this must be balanced against the risk of interference between the phages, that cross-resistance arises, or that the individual dose of each phage becomes lower. However, it has been shown that some phages produce depolymerases with the ability to degrade biofilm and that this can potentially lead to synergies between phages (90–92). Such synergies can compensate for the disadvantages and motivate the use of cocktails, especially as biofilm is a major problem in many infections.

### Development of evaluation methods

Optimisation would not be possible without the development of new quantitative methods that allow for precise monitoring of pharmacological parameters of importance for phage therapy. These methods must be applicable to quantify CFUs and PFUs in diverse tissues after delivering different formulations of phages by different routes (93). In animal experiments, it is fairly easy to determine phage and bacteria titres at endpoints, and sometimes quantitatively during treatment. Alternative techniques such as bioluminescent imaging of phages and bacteria in live animals might supply more detailed information of importance for optimisation of later clinical trials in humans. Imaging of phage infection *in vivo*, e.g. during phage therapy of mice, has not been carried out, although the technique has been established (94).

Monitoring of infection parameters is much easier with skin infections, where direct counts of titres can be performed (13). It can, however, be demanding since sampling must be carried out at short intervals and in a quantitative way. Thus, there is an urgent need for faster and easier methods for phage quantification. If the PK and PD of such treatments become reproducible, and if the bacterial titres after treatment are negligible, phage therapy could become an accepted treatment option. However, if a successful result of phage therapy of a topical infection cannot be predicted, it is hard to understand how treatment of other more complicated infections should be planned.

## Conclusion

When comparing outcomes of antibacterial treatment, administration of antibiotics often results in complete infection clearance, whereas the results of phage treatment show variation from no effect at all to significant elimination of the infecting bacterium. The goal of phage therapy research should be to reduce that variation in efficiency, i.e. increase the number of cases where the bacterial infection completely clears to substantially higher levels. Treatments that could show the same good and reproducible results as treatments with antibiotics have done would most likely lead to increased interest and enable the development of alternatives. However, the list of factors limiting phage therapy is quite long.

Much of the phage therapy research reported so far has been descriptive, typically showing the ability of a phage to infect a particular bacterial strain, and often not in quantitative terms, advocating the particular phage for therapeutic purposes. Such reports will still have some value in the future, but it is desirable that phage therapy research moves on to study *how* phage therapy works under different conditions rather than just *if* it works or not, e.g. the cases where phage therapy fails are far more interesting than reports of the sheer percentage of successful cases. It is apparently possible to get phage therapy to work, but single successful cases have shown that it takes extraordinary efforts (95,96), and it is currently difficult to amass such efforts on a broader scale.

The special pharmacokinetics and pharmacodynamics of phage therapy need to be studied in depth and phage properties as well as methods outlined that maximise the number of successful treatments. Data on a relevant number of bacteria causing serious illness, which have been proven *in vitro* to be successfully and effectively eliminated by phages, are highly warranted. Some strains will be resistant to particular phages, and many phages would be needed to cover all bacterial strains. The number of relevant bacterial species and strains to be selected as targets for phage therapy depends, however, heavily on the financial resources allocated. The primary focus should undoubtedly be on multi-resistant bacteria, severe infections, and those causing a high societal burden. Phages should be optimally selected by showing high adsorption rate, large burst size, and, less important, short latency time on at least one bacterial strain. In addition, it might be possible to find phages having a broad host range with comparable EOPs on many strains for some bacterial species.

The establishment and development of collections of phages for therapy may be faster than the development of a new antibiotic, but too slow within a local context (country). Although data on the efficacy and host range of phages in many cases are published, available data are varying, as are the phages themselves. There is a need for a centralised initiative on the collection, standardisation of methods (e.g. purification methods), and quantitative evaluation of phages suggested for therapy. Initiatives have, however, been taken recently to coordinate resources and share phages and information from research institutions around the world (97–99).
